# Co-inheritance of glucose-6-phosphate dehydrogenase deficiency mutations and hemoglobin E in a Kachin population in a malaria-endemic region of Southeast Asia

**DOI:** 10.1371/journal.pone.0177917

**Published:** 2017-05-22

**Authors:** Zeshuai Deng, Fang Yang, Yao Bai, Lijun He, Qing Li, Yanrui Wu, Lan Luo, Hong Li, Limei Ma, Zhaoqing Yang, Yongshu He, Liwang Cui

**Affiliations:** 1 Department of Cell Biology and Medical Genetics, Kunming Medical University, Kunming, Yunnan Province, China; 2 Department of Pathogen Biology and Immunology, Kunming Medical University, Yunnan Province, China; 3 Department of Histology and Embryology, Kunming Medical University, Yunnan Province, China; 4 Department of Entomology, The Pennsylvania State University, Pennsylvania, United States of America; Centro de Pesquisas Rene Rachou, BRAZIL

## Abstract

Glucose-6-phosphate dehydrogenase (G6PD) deficiency and hemoglobin E (HbE, β^26 Glu-Lys^) are two common red cell disorders in Southeast Asia. G6PD deficiency produces hemolytic anemia, which can be triggered by certain drugs or infections. HbE is asymptomatic or is manifested as microcytic, minimally hemolytic anemia. The association between G6PD deficiency and HbE is little understood. This study aimed to investigate G6PD deficiency and HbE in a Kachin ethnic group in the China-Myanmar border area. G6PD enzyme activity was measured using a quantitative G6PD assay, G6PD variants genotyped by the SNaPshot assay, and an HbE gene mutation identified by an amplification refractory mutation system and subsequently confirmed by using a reverse dot blot hybridization assay from 100 unrelated individuals in the study area. G6PD enzyme activity ranged from 0.4 to 24.7 U/g Hb, and six males had severe G6PD deficiency (<0.12–1.2 U/g Hb), while six males and 12 females had mild G6PD deficiency (>1.2–4.5 U/g Hb). Among the 24 G6PD-deficient subjects, 22 (92%) had the Mahidol 487G>A mutation (12 male hemizygotes, one female homozygote, and nine female heterozygotes), while the G6PD genotypes in two female subjects were unknown. HbE was identified in 39 subjects (20 males and 19 females), including 15 HbEE (seven males and eight females) and 24 HbAE (13 males and 11 females). Twenty-three subjects co-inherited both G6PD deficiency and HbE (22 with HbAE and one with HbEE). Whereas mean Hb levels were not significantly different between the HbA and HbE groups, G6PD-deficient males had significantly lower Hb levels than G6PD-normal males (*P* < 0.05, t-test). However, it is noteworthy that two G6PD-deficient hemizygous males with HbAE were severely anemic with Hb levels below 50 g/L. This study revealed high prevalence of co-inheritance of G6PD deficiency with HbAE in the Kachin ethnicity, and a potential interaction of the G6PD Mahidol 487G>A and HbAE in males leading to severe anemia. The presence of 6% males with severe G6PD deficiency raised a major concern in the use of primaquine for radical cure of vivax malaria.

## Introduction

Genetic disorders of red blood cells (RBCs) are important public health issues in Southeast Asian populations, among which thalassemia and glucose-6-phosphate dehydrogenase (G6PD) deficiency are two main erythrocyte disorders with clinical symptoms [1,2]. G6PD deficiency produces hemolytic anemia, which can be triggered by certain drugs or infections. Hemoglobin E (HbE) is a β-chain structural variation at the globin gene with a codon 26 G to A mutation, and is one of the most common hemoglobin (Hb) variants with a reported frequency of 50–70% in Southeast Asian populations and 10–25% in southwestern China [3–5], suggesting that this mutation might have originated in Southeast Asia. The incidence of HbE varies from different regions and among different ethnic groups. Some of the mechanisms underlying protection against malaria have been put forward to explain the variation frequency of hemoglobinopathies in malaria-endemic regions [6]. Hb-inherited disorders such as thalassemia, sickle-cell trait, HbC, and HbE provide significant protection from severe malaria syndromes [7]. However, recent reports from northern Thailand showed that the presence of HbE does not have an effect on *in vitro* infectivity and proliferation of *Plasmodium falciparum*, formation of hemozoin (the malaria pigment) [8], and cerebral malaria [9]. The distribution of differential HbE prevalence was attributed to ancestral genetic drift and admixture. Thus, the genetic fitness of HbE in Southeast Asia remains uncertain as compared with that of the HbA/S in Africa [10,11].

G6PD deficiency is widespread across malaria-endemic regions and is the most common human hereditary RBC enzyme defect disorder in Southeast Asia. G6PD gene mutations reduce enzyme activity, leaving RBCs vulnerable to oxidative stress. A number of studies provided evidence about the association of G6PD deficiency with protection against malaria, which explains the high prevalence of G6PD deficiency in malaria endemic areas [12–14]. Meanwhile, G6PD-deficient individuals are at risk of hemolysis when exposed to 8-aminoquinoline drugs such as primaquine and tafenoquine, which are needed for radical cure of vivax malaria. The high prevalence of G6PD deficiency in endemic countries and the lack of point-of-care diagnostics for G6PD deficiency hinder the wide use of primaquine for treatment of vivax malaria [15].

Malaria remains one of the most life-threatening infectious diseases in the tropics, and almost half a million people die from malaria each year. In the Greater Mekong Sub-region (GMS) of Southeast Asia, malaria transmission is heterogeneous with most of the cases occurring along international borders [16,17]. At the China-Myanmar border area, malaria transmission has been significantly reduced on the China side as China is aiming for malaria elimination by 2020, whereas vivax malaria continues to be relatively prevalent and cause sporadic outbreaks on the Myanmar side. Along many international borders of the GMS are the homes of a large number of ethnic minorities and hill tribes. The prevalence of G6PD deficiency among these populations present a major challenge for eliminating vivax malaria in these border regions. For example, among the Kachin ethnic groups, G6PD deficiency is present in more than 20% of the population [18]. The Kachin ancestors lived in Central Asia (probably east or northeast of Tibet) and migrated gradually to Southeast Asia. Nowadays the Kachins largely inhabit northern Myanmar, India and along the China-Myanmar border area. In addition to G6PD deficiency, HbE is supposed to occur at a relatively high frequency in the China-Myanmar border region, suggesting that co-inheritance of these two genetic disorders might be present in the local populations. Thus, this study aims to determine the prevalence of these two genetic disorders and explore their relationship in the Kachin population.

## Materials and methods

### Subjects

A total of 100 “healthy” unrelated subjects—comprising 53 males and 47 females, and aged 4–75 years living in villages near the Laiza township in northeast Kachin State of Myanmar along the China-Myanmar border—were recruited to the study in August 2015. The inclusion criteria were: Kachin ethnicity, unrelated to each other, women who were not pregnant, healthy at the time of enrolment (no malaria infection by microscopic examination of blood smears and asymptomatic for blood disorders), and those able to provide consent/assent. The exclusion criteria were: other ethnicities, pregnant women, not healthy, and those unable to consent/assent. Written informed consent was obtained from adult and children, while assent was also obtained from children 7–18 years. A total of 120 people were approached; five people were excluded because they belong to other ethnicities and 15 declined to participate. After obtaining written informed consent/assent, a venous blood sample (approximately 1–2 ml) was drawn from each adult participant, while a finger-prick blood sample (~200 μl) was obtained from each child participant. The blood sample was put into an EDTA-filled tube, kept on ice, and used for measuring G6PD activity within 12 hours. Hb concentrations were measured by the impedance method using an automated hematological analyzer (TEK-II Mini, China). Hb thresholds used to define anemia are <130 g/L in men and <120 g/L in women according to the World Health Organization (WHO) criteria. Hb levels at 80 –< 110 g/L and < 80 g/L are classified as moderate and severe anemia, respectively.

### G6PD assay

Quantitative determination of G6PD activity was performed using a commercial kit (Trinity Biotech, St. Louis, MO, USA) according to the manufacturer’s instructions. Quality control was performed on each day of testing using G6PDH controls manufactured by Trinity Biotech. Three control levels [deficient level (catalog no. G5888), intermediate level (catalog no. G5029), and normal level (catalog no. G6888)] were analyzed before the test panels and were considered to be valid if control values fell within the given ranges. For this assay, the cut-off value for G6PD deficiency was set at 4.5 U/g Hb, with those having G6PD activity higher than 4.5 U/g Hb considered to be normal.

### Genotyping G6PD variants

Genomic DNA was extracted from whole blood using the Takara DNA Kit (Takara Biotechnology, Japan) according to the manufacturer’s instructions. Genomic DNA was eluted in 80 μL of water and stored at -20°C until genotyping. Common G6PD variants, including 11 common G6PD variants, were genotyped using the SNaPshot assay described previously [19].

### Determination of the HbE genotype

The HbE mutation was determined using the amplification refractory mutation system PCR described previously [20]. Identification of HbE mutation was performed using the allele-specific PCR primer pair βES (5′-CGTGGATGAAGTTGGTGGTA-3′) and βEA (5′-TCCCATAGACTCACCCTGAA-3′), which produces a 400-bp fragment. Two additional primers [5′-CAATGTATCATGCCTCTTTGCACC-3′ (Control A) and 5′-GAGTCAAGGCTGAGAGATGCAGGA-3′ (Control B)] were included to produce an 861-bp fragment of the globin 3′ UTR and exon 3 to serve as an internal amplification control. The multiplex PCR reaction mixture (25 μl) contained TakaraTaq^®^ Master Mix (2×PCR Buffer, 25 mM MgCl_2_, Taq DNA polymerase, and 200 μM dNTPs), 1 μg genomic DNA, and 15 pmol of each of the four primers mentioned above. The PCR procedure included 3 min of heating at 94°C, and 30 cycles of 94°C for 1 min and 67°C for 1.5 min in a DNA T100^™^ Thermal Cycler (BioRad, Hercules, USA). The amplified DNA product was analyzed in a 1.5% agarose gel and visualized under UV light after staining. Each amplification reaction included an HbE positive sample and a healthy control.

### Reverse dot blot hybridization to analyze β thalassemia genotypes

Seventeen known β-thalassemia genotypes most commonly seen in the Chinese and Southeast Asian populations [41/42M (-TTCT), 654M (C→T), -28M (A→G), 71/72M (+A), 17M (A→T), βE M (G→A), 31M (-C), 27/28M (+C), IvsI-1M (G→A, G→T), 43M (G→T), -32M(C→A), -29M (A→G), -30M (T→C), 14/15M (+G), CapM (-AAAC), IntM (T→G), and IvsI-5M (GRC)] were analyzed by PCR reverse dot blot hybridization (Yishengtang Biological Products Co., Shenzhen, China). The assay was performed according to the manufacturer’s protocol. PCR amplification products were hybridized to the test strips containing allele-specific oligonucleotide probes, which covered 17 β-thalassemia genotypes.

### Statistical analysis

G6PD enzyme activity data and Hb concentration were entered in a Microsoft Excel spreadsheet. Statistical comparison of G6PD enzyme activity and Hb concentration was done using the GraphPad Prism 6.0 statistical software. Differences in G6PD enzyme activity and Hb concentration individuals were compared using the t-test with *P*<0.05 considered statistically different.

## Results

### G6PD enzyme activity and identification of G6PD mutations

**Fig 1:**
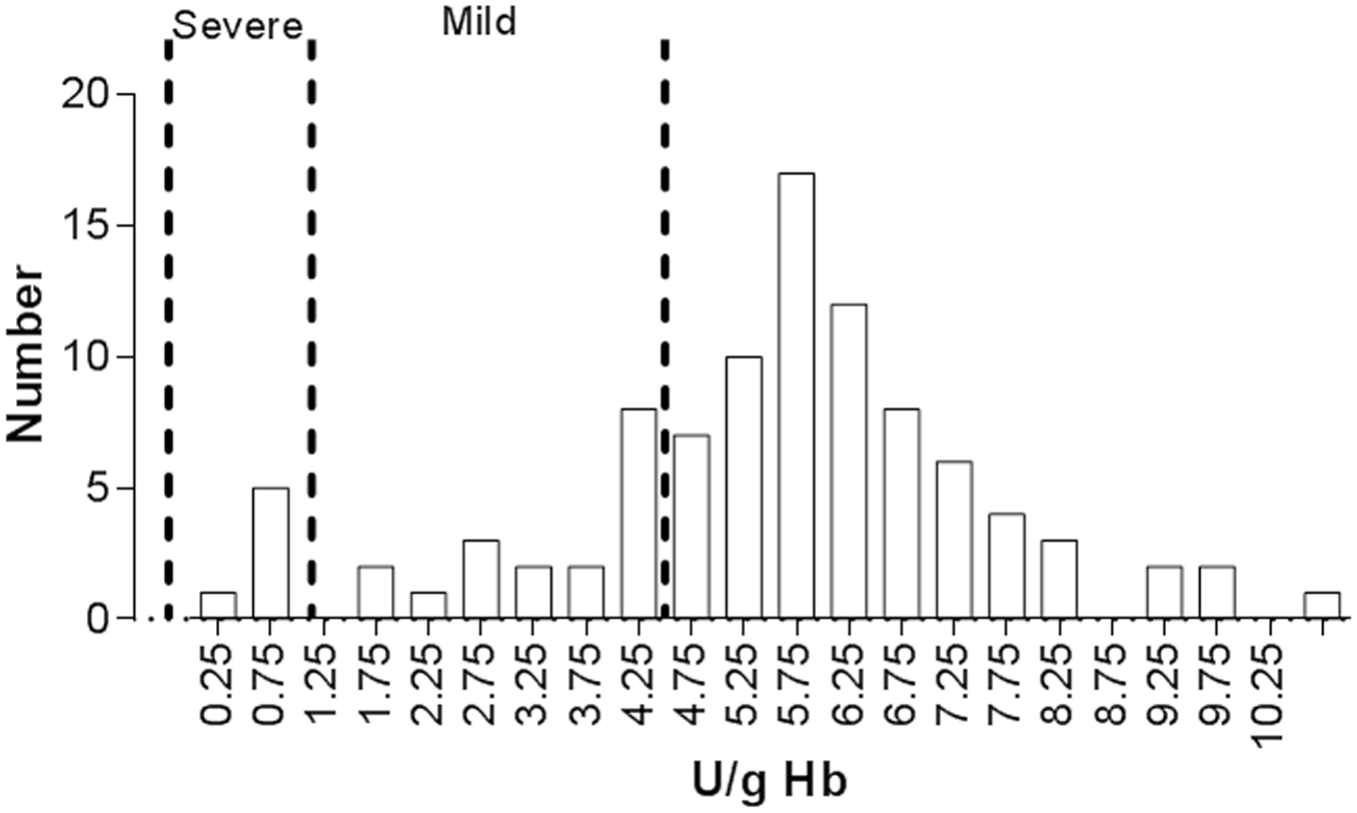
Distribution of the G6PD enzymatic activity values (U/g Hb) in 100 individuals of the Kachin ethnicity. These with enzyme activity at 1.2–4.5 U/g Hb were considered mildly G6PD deficient, while those with enzyme activity below 1.2 U/g Hb were considered severely G6PD deficient based on the population mean of G6PD enzyme activity at 12.7 U/g Hb.

The mean G6PD enzyme activity of all participants was 5.7 U/g Hb, ranging from 0.4 to 24.7 U/g Hb (S1 Table). Twenty-four of the participants (12 males and 12 females) had G6PD levels below the cutoff value of 4.5 U/g Hb and were considered to be G6PD deficient (Fig 1). With the test used, the mean G6PD activity in normal females of the study population was determined to be 6.7±1.8 U/g Hb while that in normal males was 6.7±3.1 U/g Hb. Differences in mean G6PD activity between normal male and female groups were not statistically significant (*P* >0.05). Six males (25%) with G6PD enzyme activity in the range of 0.12–1.2 U/g Hb were considered to have severe deficiency, whereas 18 subjects (75%, 6 males and 12 females) with G6PD enzyme activity in the range of 1.2–4.5 U/g Hb were considered to have mild deficiency. In the G6PD-deficient hemizygous males, mean enzyme activity was 2.2±1.8 U/g Hb (0.4–4.1 U/g Hb), whereas mean enzyme activity in the G6PD-deficient female group was 2.8±0.9 U/g Hb (1.3–4.3 U/g Hb). G6PD activity was significantly lower in deficient groups than in the normal groups (*P* < 0.05). However, there was not a significant difference in the mean G6PD activity between the G6PD-deficient female and male groups (*P* > 0.05). According to the WHO classification, which defines G6PD enzyme activity levels in the 0.12–1.2 U/g Hb (>1–10% of the normal enzyme activity level) as class II or severe deficiency, the six G6PD-deficient males should belong to this class.

The 24 G6PD-deficient subjects were genotyped for the 11 common G6PD variants occurring in Southeast Asia using the Snapshot assay. Consistent with the earlier finding [16], the majority (22/24) of them carried the G6PD Mahidol variant (487G>A), including 12 male hemizygotes, 1 female homozygote, and 9 female heterozygotes. Two (8%) females had unknown G6PD genotypes. All six aforementioned males with severe G6PD deficiency had the G6PD Mahidol 487G>A mutation. This result showed that the G6PD Mahidol variant (generally considered to be benign or to only cause moderate levels of deficiency) could also cause severe G6PD deficiency in males (Fig 1).

### β-thalassemia genotypes and Hb levels

Using an allele-specific PCR for HbE (S1 Fig), a total of 39 (39%) participants including 20 males and 19 females were found to carry the HbE alleles. To identify whether these 39 HbE individuals carried additional β-thalassemia genotypes, they were analyzed for the 17 known β-thalassemia genotypes using a PCR reverse dot blot hybridization assay (S2 Fig). This analysis only identified HbE mutations in the population, while the rest of 16 β-thalassemia genotypes were not found. HbE heterozygotes had two hybridization spots in the N (wild-type) and M (mutation) probe blots (S2 Fig), whereas homozygotes had one hybridization spot in the M (mutation) probe blot (S2 Fig). The 39 subjects carrying the HbE alleles were identified to be 15 HbE homozygotes and 24 HbE heterozygotes.

### Co-inheritance of G6PD deficiency and HbE

In the study population, co-inheritance of G6PD deficiency and HbE occurred at 23% (Table 1). Twenty-two G6PD-deficient subjects carrying the G6PD 487G>A allele (12 male hemizygotes, one female homozygote, and nine female heterozygotes) were HbAE heterozygotes, whereas the two females with unknown G6PD genotypes were with HbAA and HbEE, respectively. Twenty-two of the 24 (91.7%) subjects co-inherited with HbE and G6PD deficiency were HbAE heterozygotes. Surprisingly, HbEE and HbAE genotypes were overly presented in the G6PD-deficient group (95.8%) as compared with those in the G6PD-normal group (21.1%, *P* < 0.005, nonparametric test, Table 1).

**Table 1:** G6PD phenotypes and HbE and genotypes in the study population (n = 100).

	G6PD Deficient (N = 24)			G6PD Normal (N = 76)		
	HbAE	HbEE	HbAA	HbAE	HbEE	HbAA
Male	12	0	0	1	7	33
Female	10	1	1	1	7	27
Total	22	1	1	2	14	60
Total with HbE (%) or HbAA (%)*	23 (95.8%)		1 (4.2%)	16 (21.1%)		60 (78.9%)

* For HbEE and HbAE analysis in G6PD-deficient and -normal groups, *P* <0.005, 95% (CI = 10.813–688.097)

### Potential effects of G6PD deficiency and HbE on Hb levels

**Fig 2:**
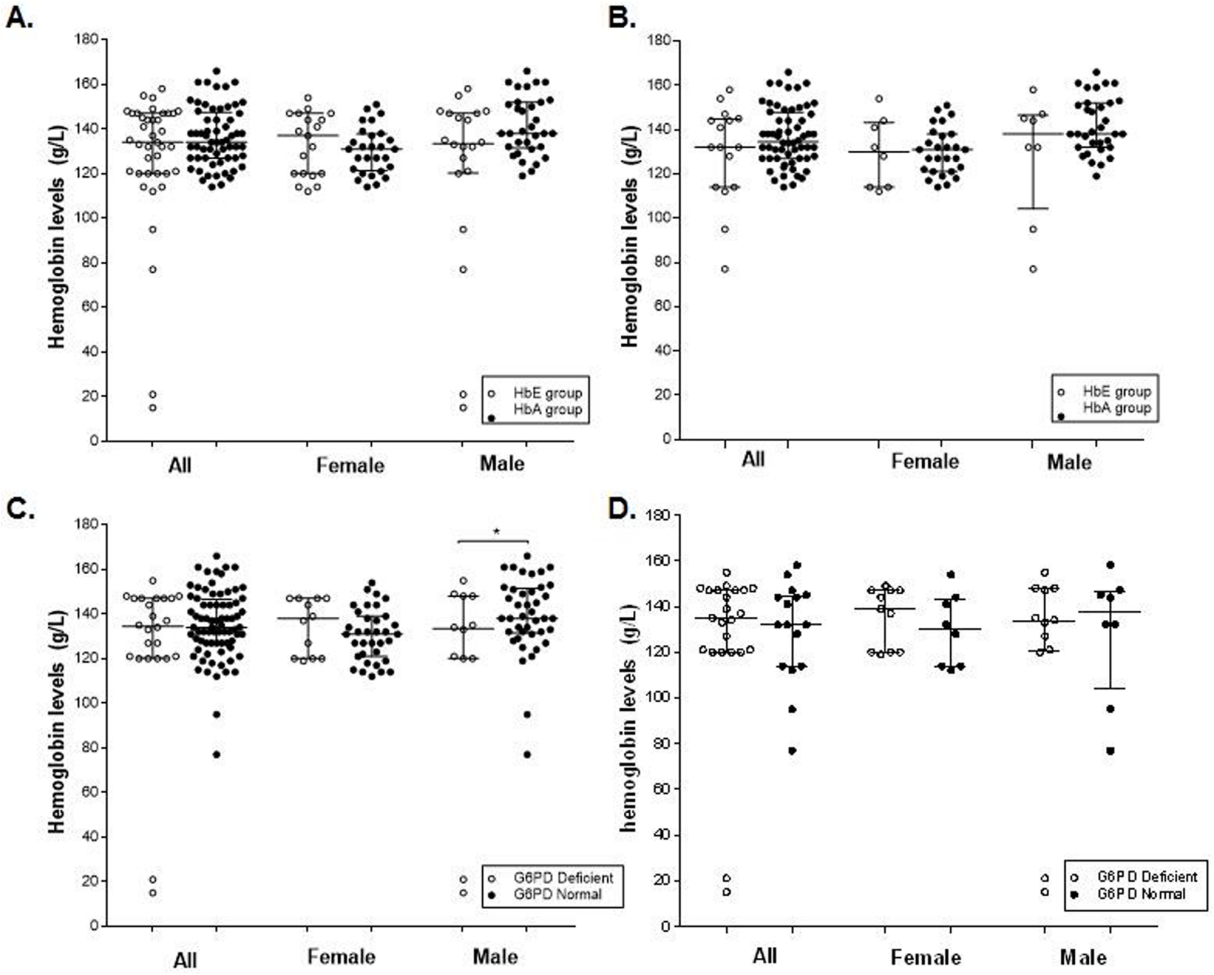
Comparison of Hb levels between different groups. **A**. Hb levels between HbA and HbE groups. **B**. Hb levels in G6PD normal individuals between the HbE and HbA groups. **C**. Hb levels between G6PD-deficient and G6PD-normal groups. **D**. Hb levels in HbE individuals between G6PD-deficient and G6PD-normal groups.

In the entire study population, there were 23 anemic people, 20 with mild/moderate anemia and three with severe anemia. The prevalence of anemia was 30.7% (12/39) in the HbE group and 18% (11/61) in the HbA group. All three subjects with severe anemia were male and in the HbE group. The mean Hb level in the HbA group (136.5±1.7 g/L, n = 61) was higher than in the HbE group (127.3±4.9 g/L, n = 39), albeit the difference was not significantly different (*P* > 0.05, t-test) (Fig 2A). The mean Hb levels were 141.5±2.3 g/L and 121.0±9.0 g/L in the HbA and HbE male groups respectively, while they were 130.5±2.0 g/L and 133.9±3.1 g/L in the HbA and HbE female groups, respectively. These levels were not significantly different from each other (*P* > 0.05). Furthermore, to determine the potential effect of the HbE trait on the Hb level without the influence of G6PD status, we analyzed the effect of HbE in G6PD normal individuals (Fig 2B). The results showed no difference in Hb levels between the HbE and HbA groups, although one male with severe anemia and one male with moderate anemia both were in the HbE group (Fig 2B).

Comparison of the Hb levels between the G6PD-normal (135.2±1.8 g/L, n = 76) and -deficient (125.8±7.2 g/L, n = 24) groups did not detect a significant difference (*P* > 0.05, t-test) (Fig 2C). Where Hb levels were not significantly different between G6PD-normal (130.6±2.0 g/L, n = 35) and -deficient (135.8±3.3 g/L, n = 12) females (*P* > 0.05, t-test), G6PD-deficient males had significantly lower Hb level (115.4±13.6 g/L, n = 12) than G6PD-normal males (139.1±2.7 g/L, n = 41) (*P* < 0.05, t-test).

Further analyses were performed to determine whether co-inheritance of the G6PD variant and the HbE allele affected the Hb levels. Among the HbE (both HbEE and HbAE) subjects, Hb levels in the G6PD-normal (129.3±5.5 g/L, n = 16) and G6PD-deficient (125.9±7.5 g/L, n = 23) groups did not differ significantly (*P* > 0.05, Fig 2D). In addition, among the HbE females, Hb levels were also similar in the G6PD-normal group (129.9±5.6 g/L, n = 8) and G6PD-deficient group (136.8±3.5 g/L, n = 11) (*P* > 0.05). However, among the HbE males, the Hb level in the G6PD-normal group (128.8±9.9 g/L, n = 8) was higher than in the G6PD-deficient group (115.8±13.6 g/L, n = 12), but the difference did not reach statistical significance (*P* > 0.05, Mann-Whitney test, Fig 2D). Nevertheless, two G6PD-deficient males with HbAE displayed severe anemia with Hb levels below 80 g/L, suggesting that co-inheritance of the G6PD deficiency and HbAE may have more severe impacts on Hb levels in males.

### Discussion

The spread and fixation of malaria-related red-cell disorders are driven by migration and selection pressure of malaria in malaria-endemic areas. Strong positive selection for malaria resistance imposed on these genes has led to high frequencies of these mutations in the human populations under selection [21], and the selective advantage has left a number of genetic variants as genome imprints over generations [22]. The malaria-resistant red-cell disorders such as G6PD deficiency and thalassemias are common in parts of Southeast Asia where malaria is endemic [23–25]. The high frequencies of G6PD deficiency linked to the X chromosome and recessive autosomal HbE disorder observed in the China-Myanmar border area suggest selection advantages in the human host, which led to their co-adaptation. Most previous studies about the influence of hemoglobinopathies on malaria focused on the HbS and HbC mutations, whereas HbE was rarely addressed. This study addressed two prevalent erythrocyte disorders, G6PD deficiency and HbE, in the malarious Kachin region of northeastern Myanmar, and revealed that these two disorders co-occur at high frequencies in the study population. A potential link between Hb levels and these two genetic RBC disorders was explored both individually and in combination.

This study further confirmed that the prevalence (>20%) of G6PD deficiency in people of Kachin ethnicity living near the China-Myanmar border was slightly higher than that determined for the Karen ethnicity residing at the Thailand-Myanmar border. In both border regions, the Mahidol type was the predominant variant. Although the Mahidol variant is historically considered as a WHO Class III G6PD deficiency and is associated with mild levels of enzyme deficiency, this study revealed that half of the hemizygous males with the Mahidol 487G>A mutation had severe G6PD deficiency. This finding echoed that by Bancone et al. (2014), who reported that the G6PD enzymatic activity of the Mahidol variant in some hemizygous males was below 10% of the G6PD activity in normal males at the Thailand-Myanmar border [26]. Given that primaquine is likely to induce acute hemolysis in these patients with severe G6PD deficiency, it is highly necessary to evaluate G6PD deficiency in malaria patients prior to prescribing primaquine for radical cure of vivax malaria.

Since oxidative stress may trigger hemolysis of senescent G6PD-deficient RBCs with reduced levels of NAPDH, severe G6PD deficiency is linked to severe hemolytic anemia induced by food (e.g., fava bean) and drugs (e.g., primaquine) [27]. Although Hb levels were similar between G6PD-normal and -deficient females, G6PD-deficient hemizygous males had significantly lower Hb level than G6PD-normal males. This result suggests that at least in males of the Kachin ethnicity, G6PD deficiency may be an important causative factor of anemia.

Worldwide, HbE is caused by an amino acid substitution in the β chain and predominates in Southeast Asia with reported high prevalence in parts of Thailand, Laos, Cambodia, India, Sri Lanka, and Malaysia [8,25,28]. Previous data from China’s Yunnan Province also showed high prevalence of HbE [29,30]. This study estimated a 39% overall prevalence of HbE in the Kachin ethnicity. Mean Hb levels did not differ significantly between the HbA and HbE groups regardless of sex, suggesting that HbE alone might not be the only reason for anemia. Of the several hypotheses put forward to explain the high frequency of HbE in Southeast Asia, one considered the result of selection by malaria. HbE is proposed to offer protection against the deleterious effects of malaria by reducing erythrocyte invasion by merozoites, lowering intra-erythrocytic parasite growth, and enhancing phagocytes of infected erythrocytes. The age of the HbE variant in the Thai population was estimated to be between 1,240 and 4,440 years ago using a simulation analysis [31]. Interestingly, this time frame coincided with that of the G6PD Mahidol variant, the most common G6PD mutation in Myanmar, western Thailand, and the China-Myanmar border, which also was under strong and recent positive selection over the past 1,500 years [32]. This highlights malaria as an important ecologic factor in the selection and maintenance of high frequencies of G6PD deficiency and HbE in the human populations in this region. In other words, the Kachins were historically located in mountain areas with restricted transportation. The high prevalence of G6PD deficiency and HbE may be the results of multiple factors in addition to selection by malaria. Genetic exchange with other neighboring groups such as the Achang ethnicity with similar levels of G6PD deficiency and the Mahidol variant as the major G6PD variant, as well as genetic drift as the result of restricted gene flow in the relatively small population, may also contribute to the high prevalence of the two malaria-related RBC disorders [30,33]. The high levels of co-inheritance of G6PD deficiency and HbE in the Kachin ethnicity deserves future studies with regard to their evolutionary histories.

This study evaluated the interactions of two red-cell disorders and their potential combinatory effect on anemia. Hemoglobinopathies and G6PD deficiency occur at high frequencies in malaria-endemic areas such as the GMS and co-inheritance of two traits is expected to be also common. In Thailand, screening of 410 thalassemia patient samples for G6PD deficiency identified 37 (9%) patients with co-inheritance of the two traits, among which 10 co-inherited HbE and G6PD deficiency [34]. Yet, co-inheritance of G6PD with α-thalassemia or HbE trait did not affect the hematological parameters or induce more severe anemia. In Bangladesh, a study of 202 individuals showed that 36% were HbAE and 8% were HbEE. Meanwhile, 34.7% and 6.4% of the population had mild and severe G6PD deficiency, respectively. It has been argued that the >6% of the individuals with severe G6PD deficiency necessitates G6PD screening before the prescription of primaquine, whereas the HbEE may lead to an increased risk of uncomplicated malaria. However, the co-inheritance of the two traits and their potential impacts on malaria were not examined [35]. Another survey of hemoglobinopathies and G6PD deficiency in various ethnic groups living in Shan State of Myanmar detected the overall frequencies of α-thalassemia, HbE and G6PD-Mahidol at 37.5, 20.3 and 17.5%, respectively, and G6PD-Mahidol and HbE co-occurred in 2.8% of the population [25]. In the present study, with the prevalence of HbE at 39% and G6PD deficiency at 24%, 22% of the study population was found to have co-inherited the HbE and G6PD 487G>A variant. Tagarelli et al. (2000) reported that in the Calabrian population in Italy the G6PD enzymatic activity of thalassemia trait groups was significantly higher than that from the normal subjects [36]. However, our study showed that most of the participants who co-inherited both traits showed low G6PD enzyme activity. This discrepancy may lie in the types of G6PD deficiencies and thalassemias involved in these studies. The early study used both α- and β-thalassemia subjects who had a high number of young erythrocytes in circulation, which have contributed to the increased G6PD activity. However, our results indicated that the HbE trait in Southeast Asia did not show a clear effect on Hb levels. Nevertheless, two G6PD-deficient males with HbAE were severely anemic with Hb levels of < 80 g/L, suggesting that co-inheritance of these two traits may have more drastic effects on anemia in males. Previous speculations on malaria as a selective agent favoring G6PD deficiency and HbE were based on evidence derived from their geographical distributions. A population genetic study further indicated that the Mahidol G6PD variant provides significant protection against *P*. *vivax* malaria [32]. Interestingly, an overwhelming proportion (22/24) of G6PD-deficient subjects in this study also carried the HbAE genotype. The reasons for such an association between G6PD deficiency and HbAE are not known, but it is possible that such a combination would offer more protection against malaria.

A major limitation of the study is that the small sample size of 100 individuals may be too small to provide sufficient statistical power and the results may not represent the true prevalence of the two RBC disorders in the total Kachin population. Nevertheless, the information on the prevalence of the two RBC disorders and the proportion of anemia estimated in this study will provide baseline information for more vigorous studies on the evolutionary histories of these disorders and their impacts on malaria.

## Conclusions

This study revealed a high prevalence of HbE (39%) and G6PD deficiency (24%) in a small population of the Kachin ethnicity. Genotyping confirmed the G6PD Mahidol variant as the predominant mutation. A G6PD enzymatic assay revealed that the Mahidol variant could be associated with G6PD enzyme activity levels below 10% of the normal level. Interestingly, HbEE and HbAE genotypes were significantly overrepresented in the G6PD-deficient group. In this area of *P*. *vivax* endemicity, the presence of severe G6PD deficiency highlights an increased risk of hemolytic anemia due to primaquine use and emphasizes the necessity of screening for G6PD deficiency before prescribing radical treatment with primaquine.

## Supporting information

S1 Table Demographic information and results of G6PD and hemoglobin (Hb) analyses. (XLS)

S1 Fig Detection of HbE alleles by allele-specific PCR. HbE allele-specific PCR produces a band of 400 bp, while an internal control of 861 bp was included in each sample. M, 1000 bp DNA Ladder; Lanes 1–6, DNA products of individual samples. Lanes 1, 2, 4 and 6 show the 861 bp PCR products from normal samples, while lanes 3 and 5 show the PCR products of 861 bp and 400 bp from samples carrying the HbE mutant allele.
(TIF)

S2 Fig Genotyping of β thalassemia genotypes by reverse dot blots. **A**. The positions of the probes blotted on the nylon membrane used for the reverse dot blot assay. Wild-type and mutant probes are denoted by N and M, respectively. **B**. Representative image of a blot from a normal person showing hybridization to the first row only. **C**. Representative image of an HbE heterozygote showing hybridization to both βEN and βEM probes in column 6. **D**. Representative image of an HbE homozygote showing hybridization only to the βEM probe in column 6.
(TIF)
