# Supplementary figures and images for: Co-inheritance of glucose-6-phosphate dehydrogenase deficiency mutations and hemoglobin E in a Kachin population in a malaria-endemic region of Southeast Asia
Source: PLoS One. 2017 May 22;12(5):e0177917. doi: 10.1371/journal.pone.0177917 (PMC5439682; doi:10.1371/journal.pone.0177917)

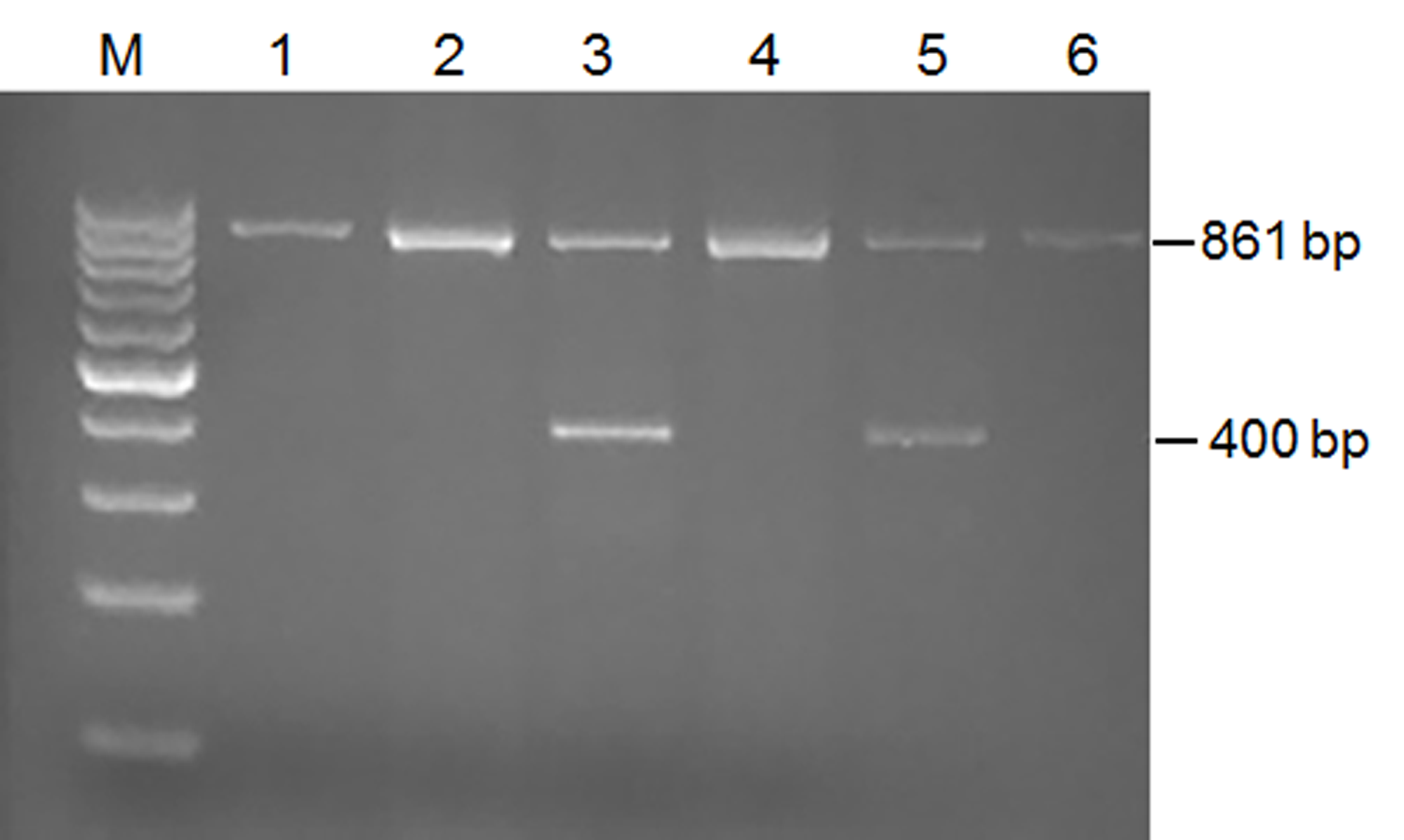

Supplement: S1 Fig — HbE allele-specific PCR produces a band of 400 bp, while an internal control of 861 bp was included in each sample. M, 1000 bp DNA Ladder; Lanes 1–6, DNA products of individual samples. Lanes 1, 2, 4 and 6 show the 861 bp PCR products from normal samples, while lanes 3 and 5 show the PCR products of 861 bp and 400 bp from samples carrying the HbE mutant allele. (TIF) [file pone.0177917.s002.tif]

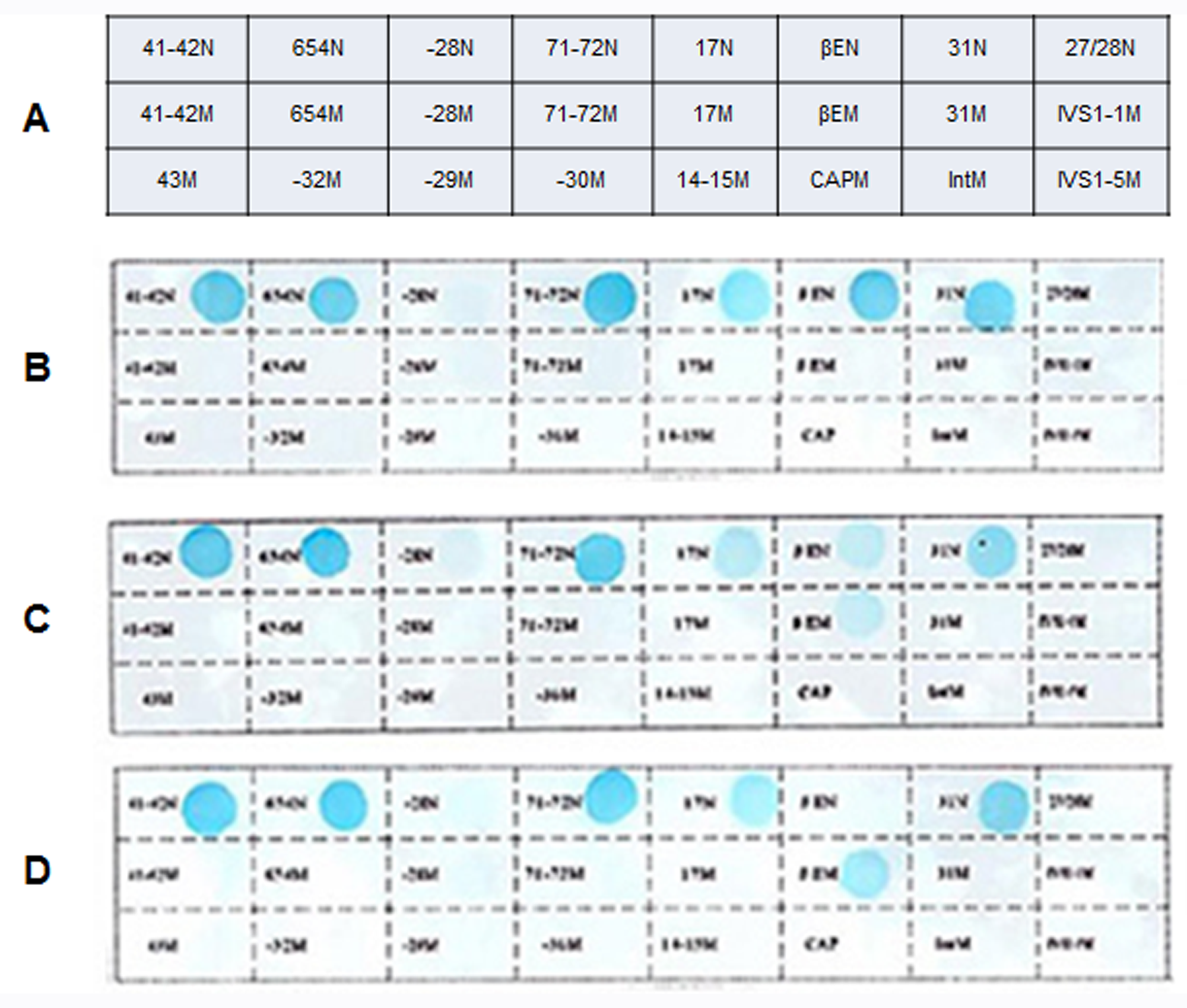

Supplement: S2 Fig — A. The positions of the probes blotted on the nylon membrane used for the reverse dot blot assay. Wild-type and mutant probes are denoted by N and M, respectively. B. Representative image of a blot from a normal person showing hybridization to the first row only. C. Representative image of an HbE heterozygote showing hybridization to both βEN and βEM probes in column 6. D. Representative image of an HbE homozygote showing hybridization only to the βEM probe in column 6. (TIF) [file pone.0177917.s003.tif]
